# Public Engagement with Romanian Government Social Media Accounts during the COVID-19 Pandemic

**DOI:** 10.3390/ijerph20032372

**Published:** 2023-01-29

**Authors:** Vasile Gherheș, Mariana Cernicova-Buca, Marcela Alina Fărcașiu

**Affiliations:** Department of Communication and Foreign Languages, Politehnica University of Timisoara, 300006 Timisoara, Romania

**Keywords:** social media, public health, engagement, COVID-19, campaign, governmental communication, Facebook, YouTube, comment analysis

## Abstract

Following international best practice examples, the Romanian government resorted to its social media accounts to disseminate information and unfold an awareness and persuasion campaign to fight the COVID-19 pandemic. The article focuses on the use of the government’s YouTube and Facebook accounts to persuade the population to get vaccinated in 2021 via video messages tailored for this purpose. The research quantified the volume of public engagement with the two waves of the persuasive campaign, finding that, while click-based reactions tended to show a positive attitude toward the campaign, the comments mainly denoted frustration, anger, and anxiety on the part of the public. Moreover, the citizens’ engagement with the campaign messages was by far lower than the number of subscribers and followers of the analyzed social media accounts. The goal of the COVID-19 public health persuasion campaign was to build support for the governmental policy and minimize the risks of the pandemic while vaccination was being rolled out. The public reaction to the governmental campaign showed that a finer and more adapted approach was necessary. This study offers a qualitative basis for informing health communication strategies of the challenges posed by social media platforms used in crisis times.

## 1. Introduction

The COVID-19 pandemic posed numerous challenges worldwide for all sectors of life. After the initial lockdown measures and recommendations for social distancing, facemask wearing, and increased personal hygiene, once vaccines became available, the World Health Organization shifted efforts toward stimulating vaccination campaigns, to contain the virus and put an end to the public health crisis that slowed down the entire planet [1]. Recommendations were issued for countries to adopt pro-vaccination measures, and governments were urged to communicate massively, timely, and via multiple channels to keep the population informed, and to persuade the public to support the measures aimed at stopping the curve of contamination and combating vaccine hesitancy.

The Organization for Economic Cooperation and Development (OECD) documents that “to succeed in the global effort to immunize billions of people as rapidly as possible, governments need to give priority to addressing issues of trust—trust both in vaccines and in the institutions responsible for the vaccination endeavor” [2]. That is why effective crisis communication is of paramount importance. The COVID-19 pandemic was a crisis that governments had to manage through “a set of factors designed to combat crises and to lessen the actual damages inflicted” [3,4]. According to the OECD 2020 Understanding Public Communication Survey, 58% of the government centers that responded to the questionnaire identified crisis communication as “the most challenging communication competency, due to human resource and coordination-related issues” [2]. At the beginning of the pandemic, some governments tried to deny the dangers that the virus was creating, portraying themselves as saviors. As time passed, most governments could not hide these dangers and resorted to different modes of crisis communication to manage the explosive situation [5]. There were countries that were successful in the vaccination campaign, while others failed, with the results depending on “the speed and scale of governmental intervention and how communities have received, perceived, and acted on the information provided by governments and other agencies” [6]. Against this background, numerous governments increased their presence on social media accounts, following a trend identified in OECD countries with government social media accounts (GSMA) serving as a source of community updates and helping governments improve emergency situations [7]. Although Romania was, at the time of this research, only an associate member of some of the OECD bodies [8], it has already started implementing OECD recommendations and has been monitored by this organization along relevant criteria, among which healthcare provisions [9]. 

Throughout the research on the topic, a persistent question is whether populations have been interested in and engaged with the social media presence of their governments [7]. There is a growing body of scientific literature exploring the use of social media during COVID-19 [10,11,12] and of government social media accounts either in one country [13,14,15,16,17], or comparatively across several countries [10,18,19]. Research ranges from the analysis of the networking sites (such as Facebook), information-disseminating platforms (such as YouTube), or microblogging services (Twitter) to mapping the field of the social media sphere, with the pandemic and governmental health crisis communication as topics [10,11,12,20,21]. The present study aims to follow the line of inquiry opened by the question of whether the public was interested in and engaged with the government social media accounts during the COVID-19 health crisis in Romania. Engagement is empirically highlighted in scientific literature through the lenses of reactions displayed by the public against the messages received via social media networks, either in light, click-based responses or in the form of textual comments, creating around the original message a field of interpretation that deserves attention on its own merit. Both types of reactions can signal acceptance or rejection of the message, interpretation, nuancing, etc., inviting content analysis for a deeper understanding of the impact produced by the posted messages.

The research team focuses only on the public’s response to the persuasive campaign conducted via GSMA in 2021 to stimulate vaccination to fight the pandemic. The research questions are as follows:

- What is the public engagement with the pro-vaccination campaign conducted via Romanian GSMAs?
- Are there differences between the public reactions to the campaign among the GSMAs used to promote vaccination in Romania?
- Are there differences between the click-based reactions of the public in comparison with the linguistic (textual) comments to the pro-vaccination campaign?

## 2. Background of the Research

### 2.1. Government Social Media Platforms during the Pandemic: Information and Persuasion

Crisis situations require increased interaction between governments and community members [20], a function that social media platforms have been shown to have the capacity to fulfill [22,23]. The need to integrate social media into crisis communication efforts has been recognized both in theory and in practice, as Timothy Coombs rightfully remarked, warning that, unless crisis managers do so, “they will fail to maximize the value communication can add to crisis management efforts” [24]. During the COVID-19 pandemic, public health communicators aimed to increase public understanding of distancing rules, to maintain the flow of information regarding the dynamics of the crisis, and to maximize the impact of governmental policy through a combination of messages and campaigns. Research shows that the extent to which the quantity and quality of information from different communication channels such as social media, government pages, and news influence public understanding of public health measures is still to be established [25]. The critical approach to the content of the messages distributed via GSMA highlights that the authorities used persuasion to a large extent to determine the population’s compliance with the distancing measures, vaccination campaigns, and policies adopted in all the phases of the pandemic [16,26,27,28,29,30]. As Oxman et al. discussed [28], while both persuading people and informing them are “reasonable goals for health communication”, a heavy emphasis during the COVID-19 pandemic fell on the persuasive aspects, aimed at determining that the population should adopt a behavior of compliance with the recommended course of action: facemask wearing, social distancing, and ultimately vaccination. Persuasion, however, is critically viewed, since it diminishes the importance of critical thinking over emotional responses [30]. Among the four typical stances of government communication through social media platforms, described by DePaula [26], namely, information provision, input seeking, online dialogue, and symbolic presentation, governments chose to provide public service announcements regarding safety, health, and undertaken measures to contain the pandemic, on the one hand, and to symbolically position the authorities as in control of the situation, on the other hand, steering toward the return to “normalcy”. Government communicators typically interpreted the public engagement with the messages transmitted via GSMA by evaluating the click-based reactions in the form of shares, likes, and comments received throughout the pandemic, considering “likes” a sui generis measure of “popularity”, “shares” as “participation” or approval [31], and “comments” as “commitment” or “engagement” [26]. However, researchers warn against such an oversimplification of the interpretation, signaling that sometimes “emojis and linguistic texts can modify the meaning of each other, and it is important to study how this interplay works” [32]. The Romanian strategic group of communicators took the common ground and automatically categorized the positively loaded emojis as supporting the campaign, as highlighted in the final report on the pro-vaccination campaign subtitled “Mission Accomplished” [33]. Such an interpretation should be accepted with caution, since extensive research proves that citizens’ engagement with the GSMA does not necessarily lead to the internalization of the messages and adoption of the desired course of action [26,28,31]. Despite the urge to adopt a critical stand, it is recognized that the use of social media platforms can positively influence awareness of public health behavioral changes and public protection against COVID-19 [21]. 

### 2.2. The Pro-Vaccination Campaign in Romania

Romania followed the same communication patterns identified in most of the countries that had to fight the effects of COVID-19 spreading across the world [34]. As a recent comparative study on the governmental responses to the pandemic showed, while some governmental attitudes and actions against the pandemic might have differed at times “ranging from the complete lockdown of a whole country or continent, as in Australia, to the open borders policy of Sweden, the frame of their communication presents more similarities than dissimilarities” [35]. Once the vaccines became available, governments actively sought to persuade citizens to undergo vaccination, as a means of escaping restrictions and returning to normal life [36]. Following the recommendations set forth by the World Health Organization to provide “individuals and communities with actionable, timely, and credible health information online and offline” and to make vaccination a pillar to combat the pandemic [1], the Romanian government appointed a National Coordination Committee on Vaccination Activities against COVID-19, placing a military doctor, colonel Valeriu Gheorghiţă, in charge of steering the vaccination. 

A study conducted by the Research Institute for the Quality of Life of the Romanian Academy (ICCV) [37] on Romanians’ reluctance to vaccination against COVID-19, identified two categories of individuals:

- The first category included persons whose reasons were lack of information, misinformation, manipulation, or disinformation (36%), as well as ignorance, stupidity, and indifference (8%) [37];
- The second category included persons who feared the adverse reactions to vaccination, including death (21%), distrust in the COVID-19 vaccine (not effective, not tested enough—17%), and general distrust in vaccination (4%); to these, the belief in the nonexistence of the new coronavirus (6%) can also be added [37].

Another study revealed that Romanians generally distrust vaccines. When asked how soon they would like to get vaccinated pending the authorization of an anti-COVID-19 vaccine, only 23% answered “as soon as possible”; 23% of the Romanians said they would get vaccinated in 2021 and 36% of them later [38]. In Romania, there are other examples of vaccination campaigns that were not very successful, such as the HPV vaccination campaign of 2008 and 2009, with a success rate of 2.57% [39].

**Figure 1:**
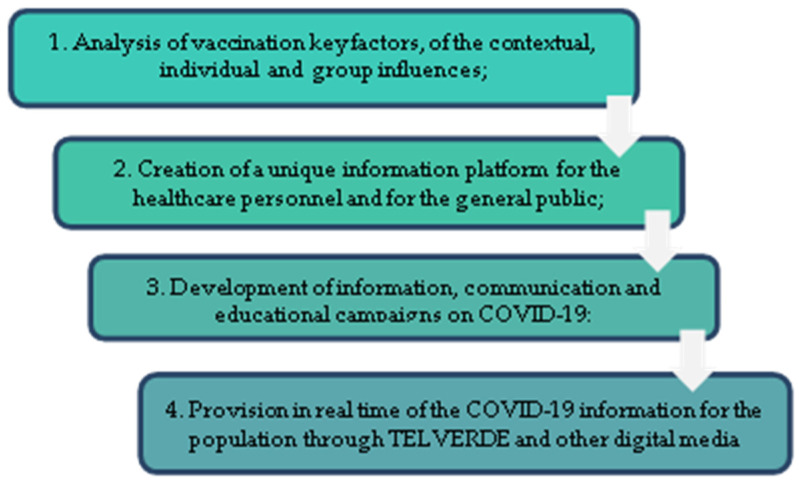
Types of interventions specified in the COVID-19 Vaccination Strategy in Romania. Graphical representation by the authors.

Therefore, it was clear that the vaccination campaign had to unfold to overcome the situation in which the population could only be persuaded through mediated communication, through media or social media channels. At the beginning of December 2020, the COVID-19 Vaccination Strategy was launched in Romania, setting out “the vision, principles, and mode of action for the administration of the vaccines authorized by the European Medicines Agency in Romania” (Figure 1).

The document specified the types of interventions and stated that “the information and communication campaigns will be carried out by involving representatives of professional associations and of patients’ associations, important figures in the cultural and educational field, and civil society representatives, and by respecting the principles of transparency and of the correct, factual, and comprehensive provision of information to the public” [40]. Other information regarding the process of communication and public information on this topic has not been disclosed.

The COVID-19 vaccination rollout in Romania was to be conducted in accordance with the specifications made in the COVID-19 Vaccination Strategy in Romania, in three phases, by population groups, as follows [40]:

- Phase 1—health and social care workers in public and private facilities, until 14 January 2021;
- Phase 2—at-risk population and the personnel in key areas of activity, from 15 January 2021;
- Phase 3—general population, from March 2021.

At the end of 2020, the first 10,000-dose batch of the COVID-19 vaccine arrived in Romania. On 27 December 2020, the first person in Romania, a nurse, was vaccinated against COVID-19. The vaccination rollout started in two hospitals in Bucharest and in eight other hospitals in Romania. This was also the moment when the Romanian authorities launched the national information platform on COVID-19 vaccination [41], to provide information and to raise awareness regarding the vaccination campaign. 

In addition to the dedicated web page, a YouTube channel [42] and a Facebook page [43] were developed to disseminate government-led initiatives to fight the pandemic. 

According to the report presented by the National Coordination Committee on Vaccination Activities (NCCVA), the engagement of the public was quite high, with up to 2.6 million engagements per week [33] through the #Rovaccinare page. NCCVA carried out the information and persuasion campaign through numerous platforms such as Facebook, YouTube, TikTok, and WhatsApp. However, the report does not differentiate between the various types of information posted on these platforms (press releases, newsletters, statistics regarding the disease rate, etc.) or among governmental messages and information from international bodies, advice from health experts, or endorsement of influencers for the vaccination campaign. 

This research focuses only on the government social media accounts established prior to the pandemic and used to promote the persuasive video messages urging the population to get vaccinated in the first half of 2021. Although the content of the video material is a topic in itself [29], the public engagement with the messages in the campaign deserves special attention, as convincingly pointed out by researchers who urge “unpacking the black box” of citizen engagement with GSMA [10,23,44].

## 3. Materials and Methods

In January 2021, Romania had approximately 16 million Internet users, representing approximately 80% of the country’s population, and 12 million active social media users, representing 62.6% of the country’s population [45]. Compared to traditional media consumers, social networks have a young audience, with communication through social networks becoming a main objective for most government and nongovernmental institutions. By 2022, the number of social media users increased to 13.3 million, representing 69.7% of the Romanian population. According to the same source, the average time spent in a day on the Internet is 7 h and 26 min, double the time spent in front of the TV—3 h and 20 min. The time spent on social networks is 2 h and 16 min, almost over 1 h more than the time spent reading the online or traditional press. The most used social network in Romania for information and interaction remains Facebook (79%), followed by YouTube (77%), while Twitter, so popular in the USA, has only 19% active users and is practically ignored by governmental communication strategists. The government’s social media presence is stronger on Facebook and YouTube, compared to all other platforms [46]. In addition to this, Romanian government communicators have experience with these platforms, with the Facebook account dating from 2012, with a reach of 287,000 followers at the time of the pandemic outbreak, and the YouTube channel dating from 2013, with a reach of 12,800 subscribers. According to the National Coordination Committee on Vaccination Activities, by the end of 2020, support was secured from Google representatives to optimize pro-vaccination content on the Internet, and the Romanian Special Transmissions Service developed the webpage https://vaccinare-covid.gov.ro/ (#Rovaccinare) as an official hub for all information concerning the pandemic, the undertaken measures to contain it, and the vaccination and the communication interventions of the officials during the health crisis. Apparently, this webpage and its associated YouTube and Facebook accounts had 35,000 subscribers [33]. 

The research team performed a web-based quantitative and qualitative analysis of the public reaction to the video content posted on the Romanian government social media accounts as part of the pro-vaccination campaign. While the Romanian GSMA abounded in a variety of video material (press conferences, information pieces, and official public declarations), this research selected only the special video productions created and disseminated as part of the pro-vaccination persuasive campaign started in December 2020 up to June 2021. The selection process dealt with the government platforms and with #Rovaccinare as the main official channels, focusing on the YouTube and Facebook accounts associated with the two entities. Some of the videos had the same content across the platforms but were labeled differently and triggered their own reactions and comments. Therefore, they were treated as separate items in the persuasive campaign.

The steps of the analysis were as follows:

1. Identification and selection of the videos posted on the Romanian government’s YouTube and Facebook channels during the pro-vaccination communication campaign;
2. Identification and selection of the videos posted on the Rovaccinare’s YouTube and Facebook channels during the pro-vaccination communication campaign;
3. Identification and evaluation of the public engagement with the videos;
4. Analysis of emotions expressed by the public in the engagement with the video messages.

**Figure 2:**
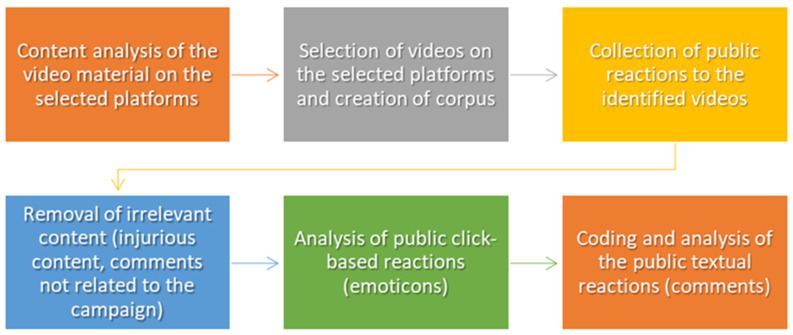
Flowchart of the research steps.

A flowchart of the research process is presented in Figure 2.

In the first stage of the pro-vaccination campaign, the Romanian government posted the video messages only on its YouTube channel (December 2020–January 2021). The same videos were offered to be distributed by traditional media, with a significant financial governmental contribution. The videos were rolled under the generic title “COVID-19 Vaccination Campaign”, having as a slogan the phrase “I’m also getting the shot” uttered by representatives of the medical profession (doctors and nurses). The promoters started with presenting the gloomy picture of losses, death, and despair caused by the virus, then stated that the solution, under the form of the vaccine, was available, and concluded with the slogan. In the second wave, roughly unfolded between May and June 2021, the government changed its strategy; new video messages were elaborated, but with endorsement from UNESCO, and with a creative concept donated for the campaign. This campaign ran under the title “COVID-19 Vaccination Campaign—Together we defeat the pandemic”, having as a slogan the nudging message “Choose to get vaccinated now!”. Actors were generically common people, longing for normal, happy lives [27]. The government communicators chose to post the video content for this campaign on the YouTube and Facebook pages, and to broadcast the content on television, but as public service messages (nonpaid, according to the National Audiovisual Council of Romania, CNA) [47]. The actors uttered emotional responses to the question “what does the vaccine contain (for me)?”. To boost the campaign, the prime minister launched on his Facebook account the challenge “what does the vaccine contain for me?”. The responses to the challenge that were promoted via the government and/or #Rovaccinare page were also included in the analyzed corpus. Furthermore, public engagement with the videos was monitored. Injurious messages or comments not related to the content were eliminated. The remaining reactions, in the form of emoticons, likes/dislikes, comments, and shares were subjected to coding and analysis. 

The corpus contains click-based reactions and linguistic comments recorded to the persuasive video material broadcast between December 2020 and June 2021 on the government’s YouTube channel and the one associated with the National Vaccination Information Platform (Rovaccinare), as well as on the Facebook pages of the two government entities. The research team retained for the analysis 19 videos posted [15,32,48,49] on the government’s YouTube channel, 26 videos on the Rovaccinare’s YouTube channel, 17 videos on government’s Facebook, and 30 videos on Rovaccinare’s Facebook. The total of 92 video materials represents the corpus of government-initiated messages, which attracted visualizations and emotional reactions, as presented in Section 4.

Following the path opened by other researchers, the reactions were analyzed according to their click-based and textual content, with a first group being formed of the emoticons and likes/dislikes, and the second one being formed of linguistic nature, from the actual comments to the content. A total of 4669 comments were retained and recorded in the corpus. While the first group allows only for an analysis along the lines of positive/negative/neutral reactions, the textual reactions allow for a more nuanced approach and require additional procedures to account for the qualitative analysis [50]. The corpus of textual reactions was split among two members of the research team, who performed the coding. A cross-checking of the coding was applied, to verify the coherence and unity in interpretation. In case of disagreement, the third researcher checked and clarified the allocation of the coding in the identified categories, ass presented in Section 4. The titles, slogans, and keywords identified in the corpus were translated into English to facilitate the understanding of the content for an international audience.

## 4. Results

In the first information/communication campaign, nine videos were identified. The campaign was launched in December 2020 under the generic title COVID-19 Vaccination Campaign, having the slogan “I’m also getting the shot”. The protagonists in this video series were specialists in the healthcare sector (medical doctors and nurses), who also held managerial positions. Each character presented in 30 s recordings the reasons why people should get vaccinated. The primary target was the medical staff in Romania, with this category being included in the first stage of vaccination in Romania. The dissemination of the videos started once the vaccine doses became available in the country. 

The second campaign “Together we defeat the pandemic! What does the vaccine contain?” was aimed at the general population. It included videos that were grouped by the research team into three categories, depending on their type of content, as follows:

1. “Together we defeat the pandemic! What does the vaccine contain?”, a campaign that included eight videos in which the characters were generically “ordinary” people, representative of different categories of population (the mother from the village, the grandmother from Muntenia (a historical–geographic region of Romania), the student, the young man, the tourist, etc.). 

2. The challenge “Together we defeat the pandemic! What does the vaccine contain?”, in which the characters in the videos (actors, journalists, and renowned doctors) responded to the challenge launched by the Prime Minister of Romania at the time, to explain what the vaccine meant to them. The aim of the campaign was to relaunch the vaccination process in Romania and to convince the population to get vaccinated. 

3. Other videos broadcast from the beginning of the vaccination campaign, in parallel with those mentioned above. These videos, produced as docudrama items, were in line with the stages and evolution of the vaccination process in Romania. The hashtags and labels of these miscellaneous videos aimed to encourage vaccination. 

A detailed analysis of the click-based reactions and comments to these videos is presented below.

### 4.1. Analysis of the Reactions on the Messages in the First Communication Campaign—COVID-19 Vaccination Campaign—I’m Also Getting the Shot (Broadcast on YouTube and Facebook)

Because YouTube ranks among the first most used social networks in Romania [51], it can be used as a tool to inform the public on issues of public interest. The Romanian government has owned a YouTube channel since 2013. At the time, it had 11,600 subscribers. The videos broadcast in the communication campaign had as a starting page the Romanian government channel, where a playlist entitled COVID-19 Vaccination Campaign was created [51]. The results (reactions) recorded by each of the videos are presented in Table 1.

The nine videos were viewed 18,850 times, with the number of views varying between 859 and 2845, resulting in a recorded average of 1713.6. The videos were uploaded on the government’s YouTube channel between 25 December 2020 and 21 January 2021. The relatively low number of views recorded on social media can be attributed to the fact that this content was already well known by the Romanian public because of its broadcast on public and private television stations as public service announcements. For the “likes” category, the variation was between six and 28. None of the nine videos was shared, and no comments were recorded, although the platform allowed posting reactions. Despite the popularity of some of the actors in these videos from other social media accounts (personal or professional), their followers did not necessarily engage with the campaign. Thus, Mihai Craiu, who won first place in the Vaccines Today Communication Challenge of 2018 and had around 300,000 followers of his Facebook account, had an impact of only 2845 views of the video featuring him in this campaign. A year later, his evaluation was that “something was missing” to make an impact with the messages during the COVID-19 pandemic urging the population to get vaccinated [52]. 

On the Rovaccinare’s YouTube channel, only two videos were identified for this first stage of the campaign, namely, the message sent by the president of NCCVA Col. Dr. Valeriu Gheorghiță and that of Dr. Adrian Marinescu from the Institute of Infectious Diseases “Matei Balș”, both with the slogan “I’m also getting the shot!”. These recordings were also broadcast on the government’s YouTube channel. Although the platform allowed reactions, no comments were recorded on the government’s YouTube channel for these videos. The same material placed on Rovaccinare attracted 49 and 12 comments, respectively, with the results presented in a special section. From the entire list of videos selected for analysis on the Rovaccinare’s YouTube channel, these were the only ones to which the possibility to add comments existed.

The presentation of the videos differed on the two analyzed entities’ social media accounts. On the government’s Facebook page, a compilation of the messages presented via the YouTube channel was posted twice, triggering the reactions presented in Table 2. Only five of the nine videos belonging to this campaign were identified on the Rovaccinare page. The total number of views was 253,000. The minimum number of views was 16,000, and the maximum was 127,000, resulting in a recorded average of 36,142.8.

The public reactions came under the form of click-based engagement (5061 emojis) and linguistic texts (1886 comments). Following the mainstream classifications [48] the reactions were classified as positive, containing the *like* (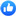), *love* (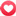), *care* (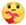), and *wow* (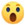) emojis, or negative, comprising the *anger* (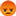) and *sad* (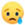) emoticons. The *haha* reaction (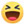) was treated as a separate category, marked *other*, since it is ambiguous in nature [15,48,49]. This classification was maintained for the whole corpus and was applied to all results referring to Facebook postings.

At a glance, it can be seen that the positive reactions represented 88.7% of the click-based reactions, while the negative ones reached 6.6% of the total. A qualitative analysis of the textual comments is presented below.

#### 4.1.1. Analysis of the Reactions to the Messages in the Second Communication Campaign—“Together We Defeat the Pandemic! What Does the Vaccine Contain?” (Broadcast on YouTube and Facebook)

Eight videos were identified in this campaign, with seven of them being posted on both YouTube channels (Romanian government and Rovaccinare). An extra video appeared on the government’s YouTube channel. A statistical overview of the reactions for the eight videos is presented in Table 3. A total of 30,979 views were recorded, with a variation of 924 to 3500 views and an average value of 2065.26. For all videos, only positive reactions were taken into account, with no negative reactions (dislikes). The number of reactions for these videos was relatively low, with a variation between seven and 38.

The Facebook accounts of the government and of the Rovaccinare platform posted only six out of the YouTube videos. Four of the videos were present on both Facebook pages, but the government’s Facebook page posted two additional videos, as presented in Table 4. The table also contains statistical data regarding the reactions triggered by the video materials. A total of 86,900 views were recorded, much more than in the case of the YouTube channels. These views ranged from 4300 to 16,000, with the average being 5690. As for the public engagement with the videos, 1939 reactions came in the form of emojis and 514 came in the form of linguistic texts. In the click-based reactions group, the positive reactions prevailed, 80.9% of the total, while the negative ones were relatively low (9.6%). 

#### 4.1.2. Analysis of the Reactions on the Messages in the Second Communication Campaign—The Challenge “Together We Defeat the Pandemic! What Does the Vaccine Contain?” (Broadcast on YouTube and Facebook)

The challenge “Together we defeat the pandemic! What does the vaccine contain?” consisted of eight videos responding to the challenge launched by the then prime minister of Romania on his own Facebook account. Sponsored by UNICEF and Google, the challenge invited influencers to find emotional arguments favoring the vaccination. All registrations were identified on Rovaccinare’s YouTube channel, with only two of them being also posted on the government’s YouTube. Table 5 shows that a total of 18,866 views were recorded, ranging between 546 and 8200. The average value was 3430.1. Only positive reactions were found (159), the number of which was also relatively low in this case, with a minimum of seven and a maximum of 40. 

The research team identified 13 videos belonging to the challenge on the Facebook pages of the Romanian government and Rovaccinare. Three videos were identified on both pages, most of them being posted exclusively on the Rovaccinare page (nine recordings). A single video was posted exclusively on the government’s page (that of the Romanian prime minister), which was, in fact, the message of the challenge addressed by Florin Cîţu to his members of the cabinet, as well as to other public figures recognized as influencers in the Romanian society. The present analysis refers only to reactions to the videos posted on the two abovementioned Facebook pages, and not to responses to the challenge posted on other accounts (such as ministerial or personal pages). As presented in Table 6, the posts belonging to the challenge cumulated 330,100 views, a value much higher than recorded in the case of the videos posted on YouTube. The variation recorded was between 1900 and 81,000 views, and the average value was 21,381.2. There were 8117 reactions recorded in the form of emojis and 1246 comments. Considering the above values, it seems that this campaign enjoyed the most success among those subjected to analysis. Moreover, the percentages recorded for the category of positive reactions (*like, love, which,* and *wow*) were clearly superior to those recorded for the negative ones (*anger* and *sad*) (90.8% vs. 4.6%).

#### 4.1.3. Analysis of the Reactions to other Messages in the Second Communication Campaign, Posted to Promote Vaccination, but under Different Slogans or Hashtags (Broadcast on YouTube and Facebook)

This category, labeled by the research team as “miscellanea” gathers the reactions to videos broadcast in parallel with the aforementioned ones (“What does the vaccine contain?”), under a variety of titles and hashtags. The government’s YouTube channel did not record such postings, while the Rovaccinare platform circulated nine videos belonging to this category. Table 7 sums up the results of the analysis. There was a relatively small number of reactions (65), all of which were of appreciation. The postings recorded a total of 4888 views, with a minimum of 267 and a maximum of 1300. The recorded average value was 543.1.

The Romanian government’s and Rovaccinare’s Facebook pages cumulated 12 videos in the “miscellanea” category. Two of them were broadcast on both Facebook pages, but the majority (seven videos) were posted exclusively on the Rovaccinare page. Three videos were broadcast exclusively on the Facebook page of the Romanian government. The number of views for this category was much higher than in the case of YouTube, with the cumulative value being 27,1100 (Table 8). The minimum number of views was 7000 and the maximum was 37,000, with the average value being 19,364.2. There were 4660 reactions recorded in the form of emojis and 736 comments. As in the previous situations, the percentages recorded for the category of positive reactions were significantly higher than the negative ones (90. 5% vs. 3.7%).

### 4.2. Analysis of the Linguistic Reactions (Comments) to the Messages in the Communication Campaigns (Broadcast on YouTube and Facebook)

Although the number of linguistic reactions was relatively small in comparison with the click-based ones, a qualitative analysis felt necessary to extract the attitudes expressed by the citizens engaging with the selected GSMAs. At first glance, the predominantly negative attitude of the public strikes the reader. Following the analysis of the valid comments (those that were related to the video or to the vaccination process), in the case of Col. Dr. Valeriu Gheorghiță, five out of the 49 comments were negative/sarcastic/ironic toward the video actor. The following reactions were also recorded: vaccination means money for other people/for the promoters (two comments), swearing/insulting the actor (two comments), vaccination means propaganda/manipulation (one comment), ironic comment toward the campaign (one comment), and technical issues related to the vaccination platform (one comment). The reactions triggered by the other video (Dr. Adrian Marinescu) were the following: negative/sarcastic/ironic comment toward the video actor (two comments), vaccination means lies and misinformation (one comment), and the idea of providing guarantees in case of vaccination (one comment). There was only one positive comment, a reaction of endorsement/support for the vaccination. 

**Figure 3:**
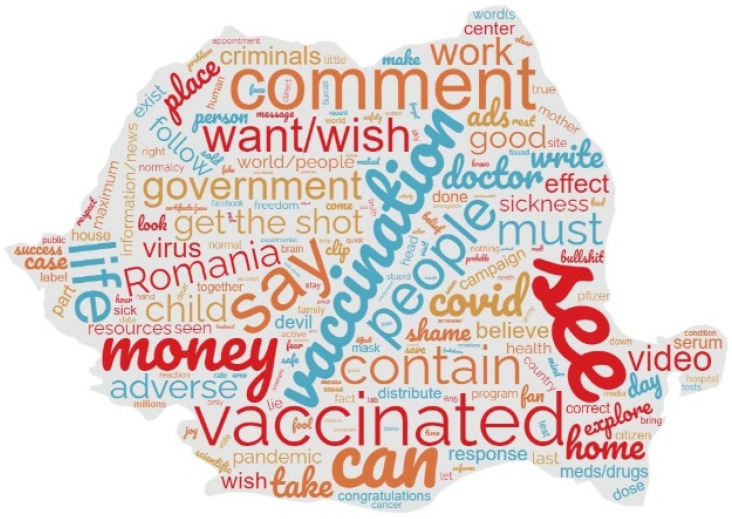
Keywords recurrent in the textual reactions to the campaign.

The messages of the two communication campaigns also recorded comments made by the citizens visiting the government’s YouTube channels and Facebook pages. As the comments were turned off on the government’s YouTube channel (https://www.youtube.com/@guvernulromaniei, accessed 2 September 2021), the only comments that could be recorded were those on the other channel on YouTube, i.e., Rovaccinare (https://www.youtube.com/@ROVaccinare, accessed 2 September 2021), and on the government’s Facebook pages (https://www.facebook.com/guv.ro and https://www.facebook.com/ROVaccinare, accessed 2 September 2021). These comments (cleaned from the nonrelated reactions) were analyzed to detect the main words expressing the public reaction (nouns and verbs). The research team translated these comments into English and processed the corpus with the help of the MAXQDA application. Words having a frequency below 10 were eliminated, and a cloud representation was employed to highlight the results, presented in Figure 3. This image shows that the public was concerned with the vaccination, the virus, and the restrictions, but also expressed anger with the government and shamed the officials, including the communicators involved in promoting vaccination.

Beyond the word frequency, a deeper understanding of the moods and attitudes of the citizens engaging with the GSMAs can be gained through a qualitative analysis of the corpus. A total of 4669 comments were collected manually from the abovementioned channel and pages and coded into categories. These categories were then classified into general themes related to vaccination. Comments including only emoticons/emojis, replies among participants in the conversation, and comments not related to vaccination were not included in the analysis. 

Therefore, the final number of comments subjected to analysis amounted to 1430. Eight general themes related to the main topic, i.e., COVID-19 vaccination communication campaigns in Romania, were revealed after data classification and are presented below, starting with those summing up the majority of comments. Each theme is subclassified and exemplified below.

#### 4.2.1. Ironic/Sarcastic/Negative Comments (43%)

This theme was the dominant one in the corpus of comments, targeting first and foremost the government (16%). Negative comments compared government officials to “Nazis” and “criminals”, with viewers wishing them “to get the shot on their tongue” as they “have lied to and humiliated the Romanian people” even more than “the Bolsheviks”. 

Negative/sarcastic/ironic comments toward the video actor also represented an important part of the comments (12.9%). When the video actors were government officials, they were “advised” sarcastically to get themselves the shot, “to even get three shots at once”, or “to get the shots in the eyes” alongside other ill-intended comments in which they were called “stupid” or “incompetent”. When the video actor was a renowned Romanian actor, the viewers were mainly disappointed that “good actors were involved” in this, asking them “how much they were paid” for doing the video. Such comments were also directed toward the vaccination campaign, which was considered by 7.7% of the viewers as “crappy”, “useless”, or “too aggressive”. Additionally, 6.4% of the comments abounded in profanities and insults.

#### 4.2.2. Vaccination Means Propaganda (15.3%)

For many Romanian viewers, vaccination meant lies and misinformation (5.4%) (“You, the government, you should get the shot and you should leave us alone! You’re making way too much out of it! Everything seems fake and you don’t show us any real proof for this vaccine”), propaganda/manipulation (4.6%) (“There’s no way vaccination will stop the pandemic! It’s the biggest global scheme ever!”), or money for other people/the promoters (4.0%) (“You, the ones doing the talking and manipulating us, how much money have you received for the videos?”). For a small percentage (1.3%) of the viewers, vaccination meant freedom, but not health (“If I have to get vaccinated to move from one place to another, it’s obvious vaccination has nothing to do with health, but with freedom”, “To get a shot only to go to the seaside or to the playground with my kids? Outrageous!”). Comments felt like part of real-life conversations, with the people writing the comments engaging in conversations where they directly reprimanded the video actor (“Liar! Shut up! Aren’t you ashamed of telling so many lies?!”).

#### 4.2.3. Vaccination and Health (12.5%)

When it comes to health, some commenters (2.6%) were annoyed that no one was mentioning the vaccine’s adverse effects (“Why don’t you tell us about the vaccine’s 15 adverse effects? About the fact that the manufacturers are not liable for these adverse effects? That it’s still under testing until 2023?”). One of the adverse effects mentioned in the comments (6.4%) was death or “slow death”, and people brought up the ones who died after they got the shot. Nevertheless, 3.7% of the commenters said that they would get vaccinated if someone took responsibility for it.

#### 4.2.4. Positive Comments and Attitudes (9.6%)

Nevertheless, there were viewers who agree with the vaccination process and even urged the others to do the same (5.9%) (“I want to live and be healthy. I can hardly wait to get the shot”, “Folks, please do get the shot! We want to return to normalcy!”). Obviously, all these positive comments were replied to with negativity and insulting comments from other viewers (“Who are you to tell us what to do?”, “We will never return to normalcy, this persecution has just started”). 

In some cases, there were also positive comments toward the video actor (0.9) (“We respect you enormously, Doctor Gheorghita!”), which, again, was received with many angry emojis and angry comments (“Are you crazy?”). The campaign was considered successful by 1.3% of the commenters (“A very fair campaign! Unfortunately, there are too many hardheaded people around”, “Finally, they have hired professionals for the advertising!”) being, of course, counteracted by sarcastic comments from people who did not believe this at all (“Are you sure you’re not referring to the Budweiser ad?”). 

Some comments discussed the benefits of vaccination (0.6%), stating that it represented one of the solutions provided by modern medicine to fight a pandemic. Vaccination meant returning to normalcy was stated by 0.9% of the comments (“We’re getting the shot. We’ll return to normalcy only through vaccination. Everyone will see that in the end”), which were replied to with sarcastic comments (“What normalcy? Does the vaccine seem normal to you?”, “You will feel sorry for yourselves in the end. We (the unvaccinated ones) will live and we will be the ones to see it”).

#### 4.2.5. Conspiracy Theory (5.3%)

The theme of the conspiracy theory was present in 5.3% of the comments. Viewers were adamant about not getting vaccinated as vaccination was seen as an experiment (2.0%) (“To force me to get vaccinated with an experimental vaccine, which has death among its adverse effects, and for which no manufacturer is held responsible, is called attempted murder!”). Around 0.8% of the commenters linked vaccination to being possessed by the devil, while 0.2% of them were afraid of vaccination as they believed gene therapy to be involved in the creation of vaccines that “changed the DNA” and “were probably made by the New World Order”. Additionally, 0.3% of the viewers feared that God would punish them if they got vaccinated.

#### 4.2.6. Anti-Vaccination (6.4%)

Approximately 3.6% of the comments strongly disagreed with being vaccinated, and 2.2% advised others to stand firm and not get vaccinated. Furthermore, 0.1% of the comments stated that the vaccine was not legal as it had not been authorized by the Minister of Health, and 0.5% considered that promoting vaccines was not legal according to the enforceable laws, even providing excerpts from these laws in the comments.

#### 4.2.7. Vaccination Process (4.4%)

In the comments, people also asked questions referring to the vaccination process (3.4%), as well as ironic ones (“Well… and when do you plan on starting to vaccinate us? Next year? If you keep this slow pace, the high-risk people won’t even be vaccinated by 2024), but also more serious ones (“When can we buy the vaccine ourselves?”). Other questions referred to technical problems related to the vaccination platform (1.0%).

#### 4.2.8. Vaccination and Discrimination (2%) 

Commenters also voiced their concern regarding the fact that vaccination was mandatory, stating that each person should decide for themselves (1.7%) and, therefore, that the vaccination should be “optional” as “it is each person’s decision what they want to do with their body”. The problem of discrimination was raised (0.3%) as there were people who could not get vaccinated due to other diseases they had. The analysis also brought forward the fact that there was a prevalence of negative comments (ironic/sarcastic/negative comments, vaccination means propaganda, vaccination and health, anti-vaccination, vaccination and discrimination, and conspiracy theory). Positive comments (positive comments and attitudes) and neutral comments (vaccination process) were present at a much lower percentage.

## 5. Discussion

This study showed that, while the intention of the communication strategists developing the pro-vaccination campaign was to trigger a massive response, the actual engagement of the viewers was much lower than for the subscribers to the government’s YouTube and Facebook accounts: 12,800 and 287,000, respectively (in 2022). The number of visualizations for the two GSMA followed this pattern, with Facebook postings recording far more views than YouTube. However, in the first campaign, aimed at persuading medical professionals to get the vaccine, Facebook was not used as a communication channel. While the first campaign was novel for the public, simultaneous with the beginning of the vaccination, it triggered more “dislikes” than “likes”, mirroring the government’s feelings of anger and anxiety in the general population, distress due to the pandemic, restrictions, and lack of perspective with respect to the length of the crisis. Despite the anticipation that “framing vaccination as a concrete, actionable strategy to reduce COVID-19 risk may help to address negative emotions, increase self-efficacy, and highlight feelings of control over reducing COVID-19 risk” [53], the abundance of negative reactions to the campaigns shows that additional calibrations of the messages were necessary for the Romanian public. The number of reactions and views of the messages increased over time, as shown by the results, especially for the challenge “What does the vaccine contain?”. This increase can be explained by the fact that, while the videos for the first two pro-vaccination campaigns were also distributed via traditional channels (mainly the television) and were produced to correspond to the communicative patterns fit for all purposes (multiplatform distribution), the responses to the challenge launched by the prime minister were placed only on the social media networks and were handled as organic, genuine social media posts. The endorsement from Google was felt in pushing the messages, as acknowledged by the NCCVA [33]. However, such data are not available for research, and it would be speculative to assess whether Google experts decisively influenced the promotion of the challenge. 

The results also show that the public engagement was more active on Facebook than on the YouTube channels, and with larger audiences on the Rovaccinare page than on the government’s social media accounts, leading to the idea that not using Facebook at the start of the communication campaign was a mistake. As for the results highlighting the quality of citizen engagement with GSMAs, in the form of click-based reactions vs. linguistic texts, a special discussion is needed.

Traditionally, “like” and “love” emojis are seen as positive responses to the Facebook postings. Marcel Danesi, in his semiotics of emoji [54] warned that, today, emojis heavily bear a phatic function, being markers of acknowledgement of contact, rather than signs of approval. In this key, the appeal to emotionally charged emojis—showing anger, disapproval, or tears adds to the interpretation that the dominant emotions triggered by the Facebook campaign were anxiety and frustration, amplified by the comments that the vaccine is not received with hope, but with skepticism and despair. 

The results of the study resonate with Zavattaro’s comment on the perils of automatically considering social media accounts as building engagement and dialogic relationships [31]. Instead, a phantom public may be created, and citizens can experience feelings of exclusion and lack of power in their relationship with GSMAs. The persuasive load of the two analyzed campaigns was obvious to the naked eye, stimulating vaccination as a reasonable goal to pursue [28]. However, the public engagement with the messages was not encouraging in terms of measuring the success of the concept behind the campaigns. At best, it showed the need for a better design and fine-tuning [28]. The Romanian communication strategists acted upon the assumption that the persuasive health communications were legitimized by the consent of the majority of the constituents [30]—be they of medical profession or members of the general public. In part, the pro-vaccination campaigns were unfolded as strategic consensual persuasion ones [55], having used both reasoned and emotional arguments regarding the necessity of the vaccination. However, the engagement of the public with the persuasive campaigns showed that positive reactions were scarce, and, in times of crises, communication strategies need constant adjustment and adaptation. The fact that the public had a negative reaction to the persuasive feature of the campaign was seen in comments such as vaccination is “propaganda”, “lies”, “experiment”, or “money for the promoters/other people”, or in highlighting the message of the Facebook campaign that “vaccination means freedom, not health”. These comments contradict the positive image derived from the analysis of the click-based responses and invite a critical assessment of the handling of the persuasive campaigns [31]. The findings resonate with research carried out in other countries on this topic in the sense that there was a gap between the governmental messages and the citizens’ communication needs [16], in terms of both content and timing of the campaigns, leading to the conclusion that a more careful listening to the public reaction is needed, especially during exceptional times such as an ongoing crisis. 

## 6. Conclusions

The findings of this research resonate with the results obtained by other researchers who studied the use of social media platforms by governments in promoting public health measures during crisis situations. It revealed the challenges of communication tasks and the need for a better tuning of the messages during the strategic consensual persuasive campaigns. The mere fact that social media platforms are welcomed by the public and that governments are encouraged to use these channels to engage citizens and foster collaborative relations with the followers does not automatically translate into approval or internalization of the messages distributed via GSMA during crises such as the recent COVID-19 pandemic. Even more so, there are studies that linked the trust/distrust in institutions and governments to vaccine acceptance [56,57,58,59,60]. 

Regarding the COVID-19 vaccination campaign, the circumstances were also not favorable as it took place against a context of low trust in the media, the health system, the doctors, authorities, and other institutions, as well as a context of an anti-vaccination attitude that had spread strongly in Romania in the previous years. As per the “Standard Eurobarometer (EB 94)—Winter 2020–2021” survey [38], conducted at the beginning of 2021, only 42% of Romanians claimed to have trust in the written press, along with 48% in the radio, 56% in the television, 37% in the online press, and 28% in social media, all of them presenting a downward trend compared to the study carried out in the previous year. Only 53% of Romanians said that they trusted the healthcare personnel, with this being the lowest rate recorded in the EU. On the other hand, 45% of Romanians said that they did not trust doctors and healthcare workers, a figure that also increased by 8% compared to the previous study. The same survey [38] also showed the fact that the main political institutions in Romania (the government, the parliament, and the political parties) recorded a descending trend related to the population’s trust, being the institutions with the lowest recorded scores. The trust in the government, for instance, dropped below 30% (29% in 2020) [38]. Other studies analyzing the public communication during the COVID-19 pandemic in Romania or similar topics [40,61,62,63,64] stressed the importance of clear, timely, and targeted-oriented messages in vaccine communication. Decision-making bodies must take into account the society’s cultural dimensions, as it is known that there are correspondences between them and the way in which people respond to such requests [65,66,67,68,69] as well as to develop more appropriate and effective responses and to prepare solutions for future crises.

Furthermore, in view of the OECD recommendations [2], in order for governments to be associated with vaccination campaigns, they must take into account the five main policy dimensions that boost people’s confidence in government institutions: responsiveness, reliability, integrity, openness, and fairness. In times when governments are looking for solutions for a more efficient use of healthcare resources, an improvement in the implementation of vaccination programs is essential, as vaccination is one of the most cost-effective interventions in the field of public health.

In addition to the information campaigns for the population, there was a need for awareness-raising campaigns to change the citizens’ behavior regarding vaccination. Although the channels used in disseminating the information varied, both new media (social media, e-mail, and blog) and traditional media (television, radio, etc.), in the absence of some studies that measured the impact of the messages among the beneficiaries of the information act, the effects may vary, and very often, the results may not produce the desired effects. The content analysis of the public reaction to the persuasive campaign showed that while, at a first glance, the Government-led messages were accepted (as proven by the large number of views and likes), a feeling of disempowerment and despair dominated the textual reactions. This attitude goes beyond the grudging and criticism identified in studies looking into public engagement with GSMAs [26], asking for additional efforts on the part of official communicators to convey meaning, and to demonstrate dialogic skills and a strong commitment to transparency. 

The lesson to learn from the approach taken by the government to tackle the pandemic is that preparedness and policies will probably set the stage for a better public response in case of future pandemics. Preparing the public to better face future pandemic threats should be seen as a mandatory prerequisite for all governments with regard to their health policies. As the next pandemic might strike any moment, governments should work toward creating strategies and plans to respond in a more informative, communicative, and timely manner, as well as toward building public trust in the health system and in their efforts.

The emergence of social media misinformation makes this task even more challenging. Therefore, teaching the public about vaccines through well-created, content-based, and culturally adapted health campaigns will definitely change the public’s perception regarding vaccination, with the main focus being on “who” and “how” the information is conveyed. 

## 7. Limitations of the Study

A series of this study’s potential limitations should also be considered. First, it would be useful to perform analyses on all the comments recorded on the pro-vaccination videos on all YouTube channels and Facebook pages, and not only on those of the Romanian government and of the National Coordination Committee on Vaccination Activities. Second, it would be necessary to carry out additional qualitative analyses to test the effects of these videos on the population. Although the messages broadcast on television and radio were the same, testing the effects among wider categories of the population could offer a wider image of the issue. Although the findings regarding Romanian governmental COVID-19-related communication cannot be extrapolated to wider contexts, it is important to point out that the present research results can be of considerable value to the research community and can help enrich the theory around health communication.

## Figures and Tables

**Table 1:** Reactions recorded for the videos on YouTube, first communication campaign (Government).

Video Title	Source	Likes	Views
1. COVID-19 Vaccination Campaign— Dr. Mihai Craiu Pediatrician	Government	19	2845
2. COVID-19 Vaccination Campaign— Dr. Beatrice Mahler Manager of “Marius Nasta” Pneumophtisiology Institute Bucharest	Government	16	2134
3. COVID-19 Vaccination Campaign— Dr. Adrian Marinescu Medical Director of “Matei Balş” Institute	Government	10	1457
	Rovaccinare	8	859
4. COVID-19 Vaccination Campaign— Dr. Simona Ionescu Commander of “Ana Aslan” ROL 2 Military Hospital	Government	6	1007
5. COVID-19 Vaccination Campaign— Dr. Valeria Herdea Family Physician specializing in Pediatrics, President of the Romanian Association for Pediatric Education in Family Medicine	Government	8	960
6. COVID-19 Vaccination Campaign— Liliana Constantin, Nurse Coordinator of the Operating Theater at Floreasca Emergency Hospital	Government	28	2560
7. COVID-19 Vaccination Campaign— Dr. Gindrovel Dumitra President of the Immunization Working Group within the Romanian National Society of Family Medicine	Government	11	1408
8. COVID-19 Vaccination Campaign— Dr. Valeriu Gheorghiță Campaign Coordinator (military uniform) Primary care physician at the Military Hospital in Bucharest	Government	15	1192
	Rovaccinare	26	2800
9. COVID-19 Vaccination Campaign— Dr. Valeriu Gheorghiță Campaign Coordinator (medical attire) Primary care physician at the Military Hospital in Bucharest	Government	13	1628
Total		126	18,850

**Table 2:** Reactions recorded for the videos on Facebook pages.

Video Title	Source	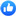	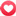	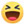	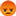	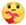	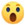	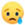	Comments	Views
Vaccination stops the pandemic and sanitary measures contain it (compilation)	Government	252	38	29	105	1			354	NA
COVID-19 vaccination campaign	Government	281	30	85	146	2	3	4	534	NA
”I’m also getting the shot!” Col. dr. Valeriu Gheorghiță	Rovaccinare	807	62	67	43	4	4	4	489	127,000
” I’m also getting the shot!” Dr. Adrian Marinescu	Rovaccinare	976	73	19	10	6	1		145	45,000
” I’m also getting the shot” Dr. Beatrice Mahler	Rovaccinare	649	25	14	7	2	1		125	16,000
What does the vaccine bring? Valeria Herdea	Rovaccinare	403	28	9	4	7		1	111	26,000
” I’m also getting the shot!” Dr. Mihai Craiu	Rovaccinare	745	83	18	8	5			128	39,000
Total		4113	339	241	323	27	9	9	1886	253,000
%		81.3	6.7	4.8	6.4	0.5	0.2	0.2		

**Table 3:** Reactions recorded for the videos on YouTube.

Video Title	Source	Likes	Views
Together we defeat the pandemic. What does the vaccine contain for a pupil?	Government	35	3600
	Rovaccinare	16	3700
Together we defeat the pandemic. What does the vaccine contain for a grandmother?	Government	38	2600
	Rovaccinare	8	955
Together we defeat the pandemic. What does the vaccine contain for a tourist?	Government	29	2700
	Rovaccinare	13	2600
Together we defeat the pandemic. What does the vaccine contain fot a grandmother in Muntenia?	Government	10	1300
	Rovaccinare	12	1900
Together we defeat the pandemic. What does the vaccine contain for a mother?	Government	7	1200
	Rovaccinare	14	1800
Together we defeat the pandemic. What does the vaccine contain for a young adult?	Government	14	1100
	Rovaccinare	18	1800
Together we defeat the pandemic. Choose to get the shot now!	Government	28	2700
	Rovaccinare	11	924
Together we defeat the pandemic—Spot Manifesto 2	Government	16	2100

**Table 4:** Reactions recorded for the videos on Facebook pages.

Video Title	Source	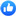	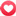	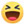	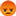	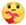	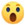	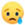	Comments	Views
What does the vaccine contain for a pupil?	Government	69	1	20	35	1	2	1	73	10,000
	Rovaccinare	374	48	6	15	5		4	45	16,000
What does the vaccine contain for a grandmother?	Government	81	6	35	18		2	5	61	10,000
	Rovaccinare	335	45	13	10	2	1	3	49	10,000
What does the vaccine contain for a tourist?	Government	45	2	21	12				53	9000
	Rovaccinare	268	26	12	10	3	3	1	75	9900
What does the vaccine contain for a young adult?	Government	35	1	24	10			1	52	4300
	Rovaccinare	135	4	2		1			21	6400
What does the vaccine contain for a grandmother in Muntenia?	Government	29	2	16	25				36	6600
What does the vaccine contain for a mother?	Government	42	1	35	36				49	4700
Total		1413	136	184	171	12	8	15	514	86,900
%		72.9	7.0	9.5	8.8	0.6	0.4	0.8		

**Table 5:** Reactions recorded for the videos on YouTube.

The Challenge on YouTube	Source	Likes	Views
Dr. Andrei Baciu, vice president of NCCVA	Government	9	1000
	Rovaccinare	7	546
Dr. Valeriu Gheorghiță, president of NCCVA	Government	40	8200
	Rovaccinare	10	909
The challenge: What does the vaccine contain? Lucian Mîndruță (TV anchor)	Rovaccinare	12	658
The challenge: What does the vaccine contain? Văru Săndel (comic actor)	Rovaccinare	15	1500
The challenge: What does the vaccine contain? Andreea Esca (TV anchor)	Rovaccinare	28	2400
The challenge: What does the vaccine contain? Victor Rebengiuc (actor)	Rovaccinare	16	1600
The challenge: What does the vaccine contain? Dr. Valeria Herdea (doctor, medical manager)	Rovaccinare	15	1300
The challenge: What does the vaccine contain? Dr. Beatrice Mahler (doctor, medical manager)	Rovaccinare	7	753
Total		159	18,866

**Table 6:** Reactions recorded for the videos on Facebook pages.

The Challenge	Source	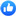	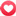	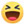	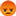	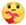	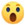	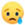	Comments	Views
The Prime Minister Florin Cîţu	Government	84	7	24	22				66	65,000
Dr. Valeriu Gheorghiță, president of NCCVA	Rovaccinare	941	99	35	27	5	4	3	134	81,000
	Government	60	2	23	37	1	2		81	3400
Assoc. Prof. Dr. Florentina Furtunescu	Rovaccinare	169	5	19	1	1			11	5400
Dr. Adrian Marinescu, doctor of infectious diseases	Rovaccinare	280	3	7	5			1	15	6900
Victor Rebengiuc, actor	Rovaccinare	937	129	16	13	9	2	3	71	24,000
	Government	131	8	35	56	1	2	6	115	4400
Andreea Esca, TV anchor	Rovaccinare	1600	159	49	47	6	2	3	208	57,000
Dr. Andrei Baciu, vice president of NCCVA	Rovaccinare	327	10	12	10	1			13	8100
	Government	40	1	11	18	2	1		18	1900
Dr. Valeria Herdea, member of NCCVA	Rovaccinare	311	32	7	4	1	1		20	13,000
Lucian Mîndruță, TV anchor and blogger	Rovaccinare	656	22	38	27	3		2	104	12,000
Dr. Beatrice Mahler, pneumologist	Rovaccinare	548	18	15	12	3			46	13,000
Dr. Adriana Pistol, director of the National Center for Surveillance and Control of Communicable Diseases	Rovaccinare	251	8	56	41	1		1	210	17,000
Dr. Raed Arafat, state secretary in the Minister of Interior, head of the Department for Emergency Situations	Rovaccinare	470	14	35	22	2	2		134	18,000
Văru Săndel, comic actor	Rovaccinare	466	19	8	13	4	3	2	50	12,000
Total		6805	517	382	342	36	16	19	1246	330,100
%		83.8	6.4	4.7	4.2	0.4	0.2	0.2	83.8	

**Table 7:** Reactions recorded for the videos on YouTube.

Miscellanea YouTube	Source	Likes	Views
Together we can change reality. Choose to get vaccinated!	Rovaccinare	7	447
“We return to normalcy. Look and move forward”	Rovaccinare	9	304
Whoever gets the shot today, dances at the castle all day!	Rovaccinare	1	293
Together we defeat the pandemic! Over 5,000,000 persons got the shot!	Rovaccinare	4	267
Together we defeat the pandemic	Rovaccinare	15	1300
Together we defeat the pandemic! Teacher	Rovaccinare	6	822
#TooMuchSuffering	Rovaccinare	15	833
You don’t have a spare life!	Rovaccinare	4	286
#TooMuchSuffering (Romani language)	Rovaccinare	4	336
Total		65	4888

**Table 8:** Reactions recorded for the videos on Facebook pages.

Miscellanea Facebook		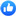	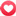	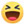	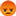	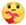	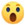	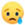	Comments	Views
We return to normalcy. Look and pay forward	Government	153	11	27	3	4	1		58	37,000
	Rovaccinare	669	94	12	1	5	1		38	37,000
The vaccine brings us #together	Government	31	1	21	22				38	7000
	Rovaccinare	142	3	1					2	7100
Together	Government	65	13	19	9	2			55	14,000
Holidays, family celebrations, concerts, laying on the beach. Today we regain them all	Government	54	2	8	15		1		37	20,000
Together we defeat the pandemic Câțu	Government	78	5	39	67		1	2	101	28,000
Together we can change the reality. Choose to get the shot! Bistrita	Rovaccinare	727	71	35	17	3	4	3	203	35,000
Together we can bring normalcy back! Vaccination marathon in Bucharest!	Rovaccinare	450	17	3	1				32	12,000
Together we defeat the pandemic!	Rovaccinare	339	12	13	2	2			11	12,000
You are the change! Be responsible! Get the shot!	Rovaccinare	444	25	9	9	1	1		45	13,000
Choose to fight the pandemic effectively!	Rovaccinare	241	20	8	14	2	1	1	29	20,000
Whoever gets the shot today, dances at the castle all day!	Rovaccinare	158	5	6	6		1		45	11,000
Green leaf, great host, turn the virus into a ghost!	Rovaccinare	316	31	70	2	3	5		42	18,000
Total		3867	310	271	168	22	16	6	736	271,100
		83.0	6.7	5.8	3.6	0.5	0.3	0.1		

## Data Availability

Not applicable.
